# The inhibition of PGAM5 suppresses seizures in a kainate-induced epilepsy model *via* mitophagy reduction

**DOI:** 10.3389/fnmol.2022.1047801

**Published:** 2022-12-22

**Authors:** Fuxin Zhong, Yunhao Gan, Jiaqi Song, Wenbo Zhang, Shiyun Yuan, Zhangjin Qin, Jiani Wu, Yang Lü, Weihua Yu

**Affiliations:** ^1^Department of Human Anatomy, Institute of Neuroscience, Chongqing Medical University, Chongqing, China; ^2^Department of Neurology, Children's Hospital of Chongqing Medical University, Chongqing, China; ^3^Department of Geriatrics, The First Affiliated Hospital of Chongqing Medical University, Chongqing, China

**Keywords:** epilepsy, seizure, PGAM5, PINK1, mitophagy, oxidative stress

## Abstract

**Background:**

Epilepsy is a common neurological disease, and excessive mitophagy is considered as one of the major triggers of epilepsy. Mitophagy is a crucial pathway affecting reactive oxygen species. Phosphoglycerate mutase 5 (PGAM5) is a protein phosphatase present in mitochondria that regulates many biological processes including mitophagy and cell death. However, the mechanism of PGAM5 in epilepsy remains unclear. The purpose of the present study was to examine whether PGAM5 affects epilepsy through PTEN-induced putative kinase 1 (PINK1)-mediated mitophagy.

**Methods:**

After the knockdown of PGAM5 expression by the adeno-associated virus, an epilepsy model was created by kainic acid. Next, the seizure activity was recorded by local field potentials before evaluating the level of mitochondrial autophagy marker proteins. Lastly, the ultrastructure of mitochondria, neuronal damage and oxidative stress levels were further observed.

**Results:**

A higher PGAM5 level was found in epilepsy, and its cellular localization was in neurons. The interactions between PGAM5 and PINK1 in epilepsy were further found. After the knockdown of PGAM5, the level of PINK1 and light chain 3B was decreased and the expression of the translocase of the inner mitochondrial membrane 23 and translocase of the outer mitochondrial membrane 20 were both increased. Knockdown of PGAM5 also resulted in reduced neuronal damage, decreased malondialdehyde levels, decreased reactive oxygen species production and increased superoxide dismutase activity. In addition, the duration of spontaneous seizure-like events (SLEs), the number of SLEs and the time spent in SLEs were all reduced in the epilepsy model after inhibition of PGAM5 expression.

**Conclusion:**

Inhibition of PGAM5 expression reduces seizures *via* inhibiting PINK1-mediated mitophagy.

## Introduction

Epilepsy is a serious chronic neurological disease characterized by long-term vulnerability to seizures, affecting over 50 million people worldwide. Although dozens of antiseizure drugs (ASDs) have been applied to epilepsy treatment in clinical practice, still more than 30% of epilepsy patients experience recurrent seizures and undesirable side effects (Lopes et al., 2015). Therefore, there is currently an urgent need to explore the pathological mechanism of epilepsy to devise new therapeutic interventions. It is now well accepted the main pathology following epileptic seizures is the loss of neuronal cells, especially in the hippocampus (Lopes et al., 2015). Interestingly, recent studies have reported that neuronal loss may be attributed to autophagy (Wu et al., 2018).

Mitophagy shows an important role in removing damaged mitochondria and maintaining normal physiological processes in cells. However, over-mitophagy has been found in the kainic acid (KA)-induced epilepsy model (Zhang et al., 2020). It is reported that damaged mitochondria accumulate abnormally in the hippocampal region of patients with epilepsy, which may be a predisposing factor for epilepsy (Liang et al., 2000). Mitochondria are the primary site of reactive oxygen species (ROS) production. A high level of mitochondrial ROS contributes to neuronal damage in seizures, which plays a vital role in the pathogenesis of epilepsy (Das et al., 2010). In addition, Mitophagy is also an essential pathway for maintaining cellular homeostasis by selectively sequestering and removing damaged mitochondria. It is reported that PTEN-induced putative kinase protein 1 (PINK1) is one of the well-known indicators related to mitophagy (Geisler et al., 2010). The primary function of PINK1 is accumulating on the outer mitochondrial membrane (OMM) of damaged mitophagy and then recruiting the E3 ubiquitin ligase Parkin to induce damaged mitochondria degradation (Meyer et al., 2017). The dysfunction of the PINK1 pathway reduces the degradation of damaged mitochondria, which usually causes oxidative damage (Kim et al., 2008; Matsuda et al., 2010; Narendra et al., 2010). Moreover, if cells cannot eliminate oxidative damage, higher levels of oxidative stress can lead to the accumulation of damaged mitochondria and defects in mitophagy (Liesa et al., 2012).

Phosphoglycerate mutase family member 5 (PGAM5) plays a significant role in regulating mitochondrial homeostasis as a serine/threonine protein phosphatase in mitochondria (Takeda et al., 2009). On the one hand, PGAM5 can protects PINK1 from the degradation of the inner mitochondrial membrane (IMM), and stabilizes PINK1 in mitochondria as well as attenuates Parkinson-like dyskinesia (Lu et al., 2014; Park et al., 2018). Also, it can also enhance oxygen consumption through the inhibition of PGAM5 in adipocytes and mitophagy (Sugawara et al., 2020). However, the role of PGAM5 in epilepsy remains unclear. It is unclear whether PGAM5 is involved in epilepsy and is associated with PINK1-mediated mitophagy. Therefore, the purpose of this study was to observe the changes of mitophagy in the KA-induced epilepsy model after the inhibition of PGAM5 to clarify the potential mechanism. These findings indicate that regulating PGAM5 may be a new method to prevent epilepsy.

## Materials and methods

### Mice

Adult male C57BL/6 J mice aged 5–8 weeks and weighing 24 g to 26 g were bred and maintained in the Laboratory Animal Center of Chongqing Medical University at specific pathogen-free conditions with a standardized environment, free eating, and drinking. Three mice were maintained in one cage. All procedures used on mice have been approved by the Commission of Chongqing Medical University for Ethics of Experiments on Animals and comply with the “Guidelines for the Care and Use of Laboratory Animals of the National Institute of Health.” The mice were randomly assigned to different groups, and the experimenter only knew the group number of mice and did not know the grouping status.

### Reagents

Rabbit PGAM5 antibody was purchased from Novus (NBP1, 92,257); Mouse PGAM5 (Santa, sc515880); Rabbit PINK1 (Proteintech, 23,274-1-AP) and translocase of the outer mitochondrial membrane 20 (TOMM20, Proteintech, 11,802-AP), translocase of the inner mitochondrial membrane 23 (TIMM23, Proteintech, 11,123-1-AP), light chain 3 (LC3, Proteintech, 14,600-1-AP); β-Actin (Proteintech, 20,536-1-AP); Mouse microtubule-associated protein 2 (MAP2, SAB, 38022) and glial fibrillary acidic protein (GFAP, SAB, 38014); Mouse ionized calcium-binding adapter molecule 1 (IBA1, Servicebio, O70200). The antibodies used in this study were used according to the instructions, and the molecular weights observed in WB were consistent with the literature (Wu et al., 2018; Wang et al., 2020; Gao et al., 2021; Brunelli et al., 2022).

### Adeno-associated virus construction and Intrahippocampal injections of virus

An AAV carried a siRNA directed against PGAM5 to reduce hippocampal PGAM5 levels (AAV-RNAi-KA group). The vehicle for the control group was Con-AAV-KA. Mice without treatment were called the control group. Control siRNA and PGAM5 siRNAs were provided by Genechem (Shanghai). Three target sequences (PGAM5 siRNA-1: CAATGTCATCCGCTATATT; PGAM5 siRNA-2: AGAAGACGAGTTGACATCC; PGAM5 siRNA-3: AGTAGAGACCACAGACATC) were assessed for excluding off-target effects. The basic Local Alignment Search Tool (BLAST,^1^) was used to verify the off-target effects. The results showed no off-target effect of siRNA - 1: CAATGTCATCCGCTATATT. Therefore, the siRNA - 1 sequence was chosen to knock down the expression of PGAM5. The mice were placed in a stereotaxic apparatus (RWD Life Science Co. Ltd., Shenzhen, China) after being anesthetized with 0.8% pentobarbital sodium through intraperitoneal injection. One microliter virus particle was injected into the DG/CA1 region of the mouse hippocampus (anterior/posterior: −2.0 mm, medial/lateral: ±1.5 mm, and dorsal/ventral: −2.0/2.5 mm). The virus injection was performed through a 5 μl syringe (Hamilton-87900, Reno, NV) with an injection rate of 0.05 μl/min. After injection, the pipette was kept in place for 10 min to minimize reflux along the injection trace. The same operation was performed on the other hippocampus.

### KA-induced mouse epileptic seizure model, behavioral video monitoring of seizure, and LFP recording

Seizures were induced by the stereotactic injection of 1.0 nmol KA (Sigma-K0250, Louis, MO) in 50 nl of saline, which was injected into the models’ left hippocampus area (2.0 mm posterior to the bregma, 1.5 mm left lateral from the midline, and 1.5 mm deep from the dura) according to the atlas of Franklin and Paxinos. The KA model was established 3 weeks after intrahippocampal injection of the virus. The chronic mouse model of spontaneous seizures induced by intrahippocampal KA injection has been widely applied (Balosso et al., 2008; Maroso et al., 2010; Chen et al., 2012; Iori et al., 2013). Two hours after the KA injection, nonconvulsive SE was terminated by diazepam.

The video monitoring process was performed as described (Kohek et al., 2021). The monitoring process was 24 h recording. Spontaneous and recurrent seizure (SRS) refers to spontaneous seizures that are demonstrable at the gross visible level under behavioral monitoring, which can be graded using the Racine scale (Aguiar et al., 2013).

A month after SE induction, the local field potential (LFP) recording was performed as previously described (Yang et al., 2018). The skull was exposed after the mice were anesthetized and fixed on a stereotaxic apparatus. Mouse bregma was used as the anchor point, and a hole was drilled at 2.0 mm posteriorly and 1.5 mm to the left of the midline by a micro electric drill. Two stainless steel screws were placed in the frontal skull for alternate ground connection and then fixed the electrode face on the stereotaxic instrument. Next, the electrode was fixed with dental cement when the electrode slowly entered the hole in the vertical direction up to 1.5 mm. Sterile cotton balls were used to secure hemostasis during drilling. The head of the awake mouse was fixed to prevent the over-behavioral state induced by LFPs changes. MAP data acquisition system (Plexon, Dallas, TX) was used to record the LFPs. The signals were filtered (0.1 to 500 Hz), preamplified (1,000×), and digitized at 4 kHz. NeuroExplorer (Nex Technologies, Littleton, MA) was used to inspect the LFPs data. The seizure-like event (SLE) is identified as a cluster of spontaneous paroxysmal discharges that lasted for 5 s or more, which is characterized by a high-amplitude spike activity above 2 times the standard deviation (SD) and a frequency above 1 Hz. All subsequent analyses (including Western Blots, imaging, etc.) were performed in animals in which behavioral experiments were previously carried out.

### Tissue collection

After anesthesia with 0.8% pentobarbital sodium through intraperitoneal injection, mice were rapidly decapitated. Twelve hippocampal tissues from each group were made into homogenates and stored at -80°C until analysis, and 6 hippocampal tissues (1mm^3^) were observed by transmission electron microscopy (TEM), and 6 hippocampal tissues were fixed with 10% formalin for 12 h. Whole hippocampal tissues were dehydrated and cut into 16 μm thick sections in a cryostat (Leica-CM1850, Germany), followed by tissue staining.

### Western blotting and coimmunoprecipitation

Western blot was performed as described previously (Biron-Shental et al., 2007). The bilateral whole hippocampus tissues of the Control group, Con-AAV-KA group, and PGAM5-RNAi-KA group were collected, and tissue proteins were extracted using lysis buffer (RIPA lysis buffer: Phenylmethanesulfonyl fluoride = 100:1, Beyotime, Shanghai, China). After centrifugation at 16,000 rpm (4°C), the supernatant was collected and stored at-20°C, followed by protein concentration determination with BCA Protein Assay Kit (Beyotime, Shanghai, China). The supernatant sample (40 μg heat-denatured protein) was separated by 12.5% SDS-PAGE gels and transferred onto 0.22 μm polyvinylidene fluoride membranes (Millipore-3,010,040,001, Billerica, MA, United States). Membranes were blocked with 5% nonfat dry milk in tris-buffered saline with Tween-20 (TBST) for 2 h at room temperature and then incubated with primary antibodies overnight at 4°C. On the next day, the membranes were washed 3 times for 5 min each with PBS saline containing Tween-20 (PBST), and incubated with PBST secondary antibody (Proteintech, SA00001-4) for 1 h at room temperature, and then washed 3 times with PBST. And bands were visualized using enhanced chemiluminescence reagents (Thermo, Marina, CA, United States) and image analysis system (Bio-Rad, United States). Relative quantitative analysis was performed using image lab software and reference proteins. The image lab software (Bio-Rad, United States) was used to analyze the optical density value of the band.

For co-immunoprecipitation, protein extracts from mice hippocampal tissue were mixed with IP lysis buffer, incubated with 4 μl rabbit IgG (Biosharp, BL003A), 10 μl PGAM5 (Santa, sc515880), and 4 μl PINK1 antibodies (Proteintech, 23,274-1-AP) overnight at 4°C, and then incubated with 40 μl protein A/G agarose beads (MCE-HY-K0202) for 2 h at 4°C. The protein bead complexes were then washed three times and collected by centrifugation. Protein samples were mixed with 1 × loading buffer (Beyotime, P0015A) and subsequently subjected to Western blotting.

### Nissl staining

Nissl staining was performed as described previously (Qin et al., 2022). Paraffin-embedded sections were dewaxed with 0.5% cresyl violet at room temperature (RT), hydrated, and stained with Nissl dye at RT for 10 min. The sections were dehydrated and sealed. Finally, each section was observed under an optical microscope (Nika A1*R. Japan), and the Nissl-positive cells were counted. For the Nissl staining, 5 images were used for statistical analysis of mice in the Con-AAV-KA group and AAV-RNAi-KA group. The number of Nissl bodies in stained tissue sections was observed in the hippocampus by light microscopy. Surviving neurons were assessed based on the presence or absence of Nissl bodies.

### TEM observation

The hippocampal tissue sections at 1mm^3^ were fixed in 3% glutaraldehyde for 1 h. The tissue sections were fixed with 1% osmium acid for 3 h and were washed 3 times with 0.1 mol/l of phosphate buffer (pH 7.4) for 5 min each time. The tissue sections were dehydrated by acetone and embedded in Epon812. Thereafter, the sections were stained with 3% uranium acetate and lead citrate (all from Sangon Biotech) at room temperature for 15 min, respectively, and were observed and photographed under a TEM (JEM-1400PLUS. Japan).

### Assay for malondialdehyde and superoxide dismutase

The levels of MDA and SOD in hippocampal tissues were measured using the MDA and SOD assay kits (Solarbio-BC0020; BC0170), respectively, following the manufacturer’s instructions, including the use of all quality controls as well as negative controls. The Cell lysis buffer was added to each hippocampal tissue in the Control, Con-AAV-KA, and AAV-RNAi-KA groups for lysing the hippocampal tissues. (Tissue weight (mg): lysis buffer volume (μl) = 1: 9, Western and IP cell lysis buffer, Beyotime, Shanghai, China). Under ice-water bath conditions, mechanical homogenization was performed to prepare the hippocampal tissue into 10% homogenate. After homogenization, the mixture was centrifuged at 10,000 g-12,000 g for 10 min, and the supernatant was taken for subsequent MDA and SOD measurements. Briefly, after the experimental procedures were performed according to the instructions, the fluorescence (excitation at 550 nm for SOD and excitation at 532 nm for MDA was measured) with a microplate reader (Thermo Fisher) was measured with a microplate reader and values were saved for offline analysis.

### Immunofluorescence staining

Immunofluorescence staining was performed as previously described (Qin et al., 2022). After anesthesia, mice were rapidly decapitated, and the brains were immediately postfixed in 4% paraformaldehyde for 24 h at 4°C. The brains were immersed in a 30% sucrose PBS solution for 48 h and subsequently sliced using a freezing microtome (Leica-CM1850); 16 μm sections were collected on glass slides. The sections were blocked with 10% goat serum (BOSTER Biological Technology) and 0.1% Triton X-100 in PBS for 2 h. The primary antibodies were diluted in 0.1% PBS added to the coverslip, and incubated overnight in a humidified chamber at 4°C. The coverslips were washed 3 times with PBS and incubated with the secondary fluorescent antibody diluted in 0.1% PBS at room temperature for 1 h. Images were captured using laser-scanning confocal microscopy (Andor-2000). The fluorescence intensity was analyzed using Image-Pro Plus software (Image-Pro Plus, v 6.0).

### Immunohistochemistry assays

Immunohistochemical staining was performed as previously described (Lopes et al., 2015). We selected frozen sections with mouse hippocampal regions for immunohistochemistry. After incubation with the primary antibody at 4°C overnight, the tissues were treated with polymer helper (ZSGB-Bio, Beijing, China) for 20 min, and then treated with poly peroxidase-anti-goat IgG (ZSGB-Bio) for 20 min. Subsequently, the tissues were incubated with diaminobenzidine Horseradish Peroxidase Color Development Kit (ZSGB-Bio) for 2 min. Counterstaining was conducted with hematoxylin (ZSGB-Bio). After each incubation step, the sections were washed with TBST for 3 times, 5 min for each time. Five images per group were used for quantitative analysis. The PGAM5 immunoreactivity was quantified automatically by digital image procedures using Image-Pro Plus software (Image-Pro Plus, v6.0). The detection threshold remained constant throughout the analysis. The percentage area occupied by PGAM5 was quantified.

### Quantitative polymerase chain reaction analysis

qPCR analysis was performed as previously described (Cao et al., 2021). The bilateral hippocampal tissues of Control group, Con-AAV-KA group, and AAV-RNAi-KA group were collected for RT-qPCR analysis. Total RNA from hippocampal tissues was prepared with TRIzol reagent according to the manufacturer’s protocol (TrizolTM Reagent, Thermo Scientific). The total RNA was reverse-transcribed to cDNA using a HiFi-MMLV cDNA first-strand synthesis kit (CW Bio, Beijing, China). qPCR was performed using GoTaq qPCR Master Mix (Promega) on the CFX96TM real-time system (Bio-Rad). The expression of the genes of interest was normalized to the levels of GAPDH. Primer sequences used for qPCR are listed below:

PGAM5: F: 5′-TGCCAATGTCATCCGCTAT-3′,
R: 5′-GGTGATACTGCCGTTGTTGA-3′.
PINK1: F: 5′-GGCTTCCGTCTGGAGGATTAT-3′,
R: 5′-AACCTGCCGAGATATTCCACA-3′.
GAPDH: F: 5′-AACTTTGGCATTGTGGAAGG-3′,
R: 5′-GGATGCAGGGATGATGTTCT-3′.

### Statistical analysis

The Kolmogorov–Smirnov and Shapiro–Wilk tests were used to test whether the data conform to a normal distribution or not when two groups were compared to each other, and an unpaired t-test was used to show the statistical significance of the data. When multiple groups were compared, one-way analysis of variance (ANOVA) was used to determine statistical significance, followed by Bonferroni correction for multiple comparisons (*post hoc* test). Non-parametric tests were used when data did not follow a normal distribution and were presented by medians and interquartile ranges. Data were expressed mean ± SD. *p* < 0.05 in this experiment was considered as a statistically significant difference, and an asterisk was used to indicate a statistically significant difference.

## Results

### Expression and distribution of PGAM5 in the epileptic mice

**Figure 1:**
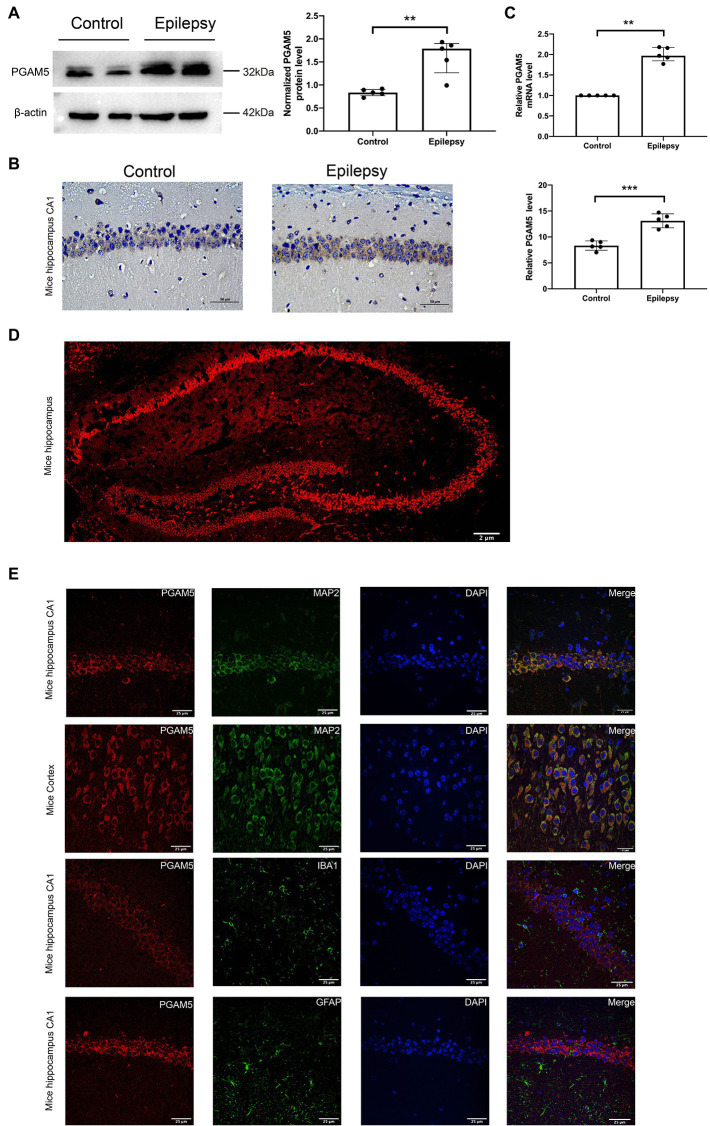
Expression and distribution of PGAM5 in the epileptic mice. **(A)** Quantitative analysis of the PGAM5/β-actin ratio, PGAM5 was increased in the hippocampus of the KA-induced epilepsy model (*n* = 5). **(B)** Distribution and expression of PGAM5 in the CA1 region of hippocampus of control mice and temporal lobe epilepsy model mice (*n* = 5). Data are expressed as Median with interquartile range. **(C)** Relative levels of PGAM5 detected by RT-qPCR analysis (*n* = 5). Data are expressed as Means ± SD **(D)** In epileptic mice, PGAM5 is expressed in the whole hippocampus. Scale bar: 2 μm. **(E)** In the hippocampus and cortex of the KA-induced epilepsy model, PGAM5 colocalizes with MAP2. Scale bar: 50 μm. **p < 0.01, ****p < 0.0001.

The bilateral whole hippocampus tissues of the Epilepsy group were obtained at 30 days after intracranial KA injection. KA-induced temporal lobe epilepsy (TLE) model recapitulates human TLE patients with typical hippocampal sclerosis (Ben-Ari, 2001). Western blot, immunohistochemistry and qPCR analysis were used to verify the relationship between PGAM5 and epilepsy by measuring the PGAM5 levels between the KA-induced TLE model and normal control mice. PGAM5 protein expression in the KA-induced TLE model was higher than that in the control group (Figure 1A, Supplementary Figure 1). Besides, the result of the immunohistochemical semiquantitative analysis showed that a higher PGAM5 level was found in the epileptic group compared with the normal control group (Figure 1B). Compare with the control group, a higher PGAM5 mRNA level was found in the epileptic group (Figure 1C). To further verify the distribution of PGAM5 in epileptic brain tissue, we performed the IF staining that showed PGAM5 was concentrated in the hippocampal CA1, CA3 and DG regions (Figure 1D). Notably, PGAM5 was colocalized with MAP2 (a marker of dendrites), and there was no distinct PGAM5 colocalization in GFAP (a marker of astrocytes) and IBA1 (a marker of microglia). PGAM5 was also expressed at high levels in scattered neurons in the cerebral cortex (Figure 1E). Therefore, it could be seen that PGAM5 is mainly expressed in neurons.

### PGAM5 inhibition mice decreased epileptic seizure activity and neuronal loss

**Figure 2:**
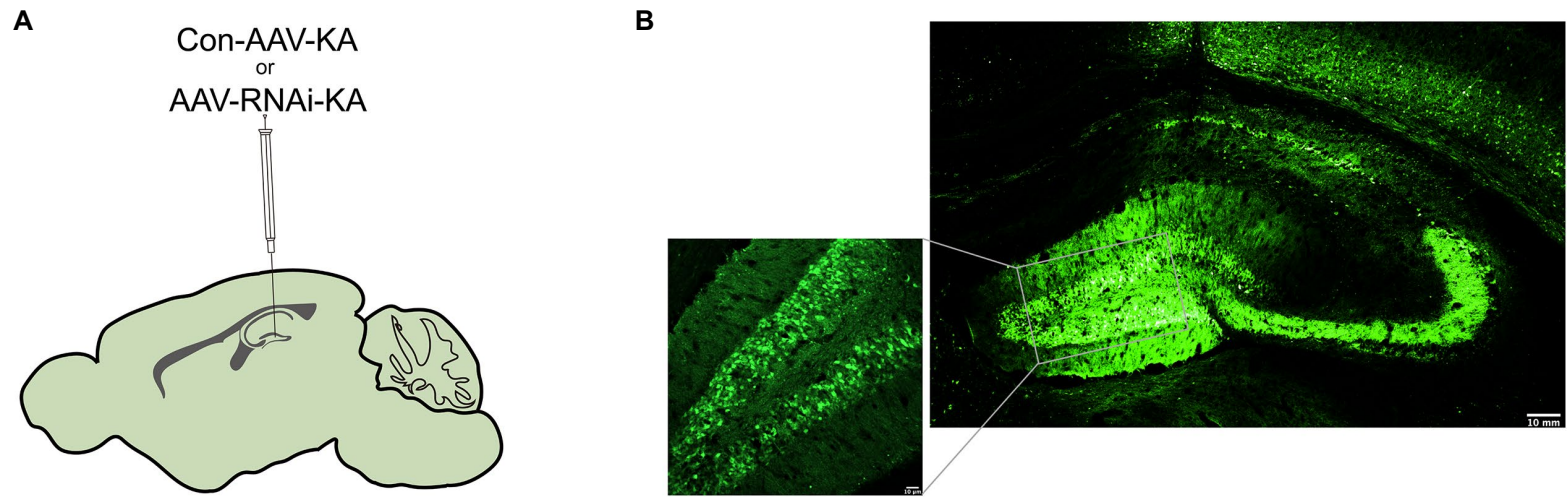
Intracranial injection of AAV with knockdown of PGAM5. **(A)** Inset of mouse hippocampal sections showing injection sites for Con-AAV-KA or AAV-RNAi-KA. **(B)** Immunofluorescence staining of AAV expression (green) in the hippocampus of mice injected with Con-AAV-KA.

Altered PGAM5 expression may be a phenomenon caused by epilepsy that indicates PGAM5 plays a causal role in seizures. Therefore, PGAM5 knockdown AAV was used to investigate whether PGAM5 level regulated epilepsy activity in the KA-induced TLE mouse model. After injecting AAV into the CA1 and DG regions of the hippocampus of mice (Figure 2A), we first used immunofluorescence for AAV expression (green) in the hippocampus of Con-AAV-KA and AAV-RNAi-KA injected mice (Figure 2B). Compared to the sham-operated groups, the knockdown efficiency of PGAM5 was 58% (Supplementary Figure 2). After 3 weeks, we created a model of epilepsy by KA injection.

**Figure 3:**
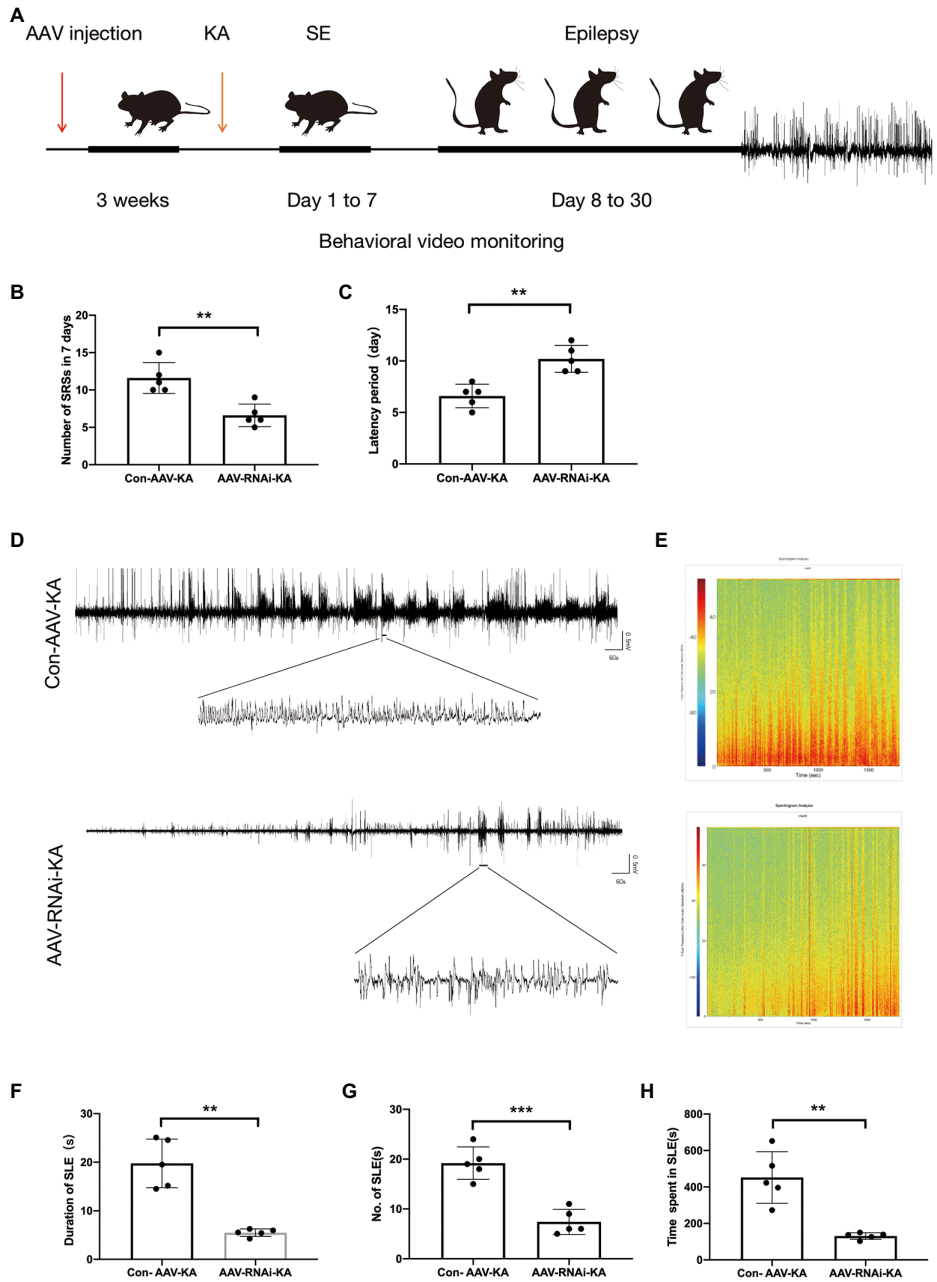
PGAM5 inhibition mice decreased epileptic seizure activity. **(A)** Graphical representation of the experimental timeline for the mouse KA injection model. Mice were injected with Con-AAV-KA and AAV-RNAi-treated mice in the hippocampus 3 weeks before KA injection. **(B)** Behavior video monitoring of mice revealed that the number of SRSs was reduced at 24–30 days in AAV-RNAi-KA group. Compared with Con-AAV-KA, the latency time of AAV-RNAi-KA was prolonged (*n* = 5). **(C)** Representative LFPs of Con-AAV-KA and AAV-RNAi-KA (*n* = 5). **(D)** Spectrum of LFP corresponding to **(B)**. **(F–H)** During 30 min, the duration of SLE, the number of SLEs and the total time spent in SLEs was reduced in the AAV-RNAi-KA group (*n* = 5). Data are expressed as Means ± SD, *p < 0.05, **p < 0.01, ***p < 0.001.

Behavioral video monitoring was used to record the seizure in mice for a month after the KA injection. We compared the grade IV and V seizure behavior of mice in each group because grade III seizure behavior and below are prone to miss. Behavior video monitoring of mice revealed that the number of seizure frequency was reduced at 24–30 days in the AAV-RNAi-KA group (Figure 3B). Compared with Con-AAV-KA, the latency time of AAV-RNAi-KA was prolonged (Figure 3C). Next, behavior may have some limitations for seizures, we recorded seizure activity and SLE by LFPs after the induction of status epilepticus (SE) to obtain a more convincing judgment. Therefore, after the video behavioral analysis in the KA model, we performed LFPs to record seizures in each group of mice. Consistent with other studies, frequent and repetitive SLEs were observed in all mice with KA injection (Figure 3A). The results showed that mice in all groups had significant epileptiform discharges (Figures 3D–E). Compared with the AAV-RNAi-KA group, the increased number of SLEs and more total time of SLEs spent in 30 min were found in the Con-AAV-KA group (Figures 3F-H).

To further explore the effect of knockdown PGAM5 on neuronal survival, we performed the Nissl staining to identify Nissl bodies in nerve cells 1 month after SE induction. In the AAV-RNAi-KA group, most of the pyramidal cells in the CA1 and CA3 regions of the mouse hippocampus were clearly demarcated and morphologically normal, and a few neurons had blurred contours. Besides, a large number of dense vertebral cells were neatly arranged, and the edges and nucleoli were clear. There were abundant Nissl bodies in the cytoplasm, and the cytoplasm was clear.

**Figure 4:**
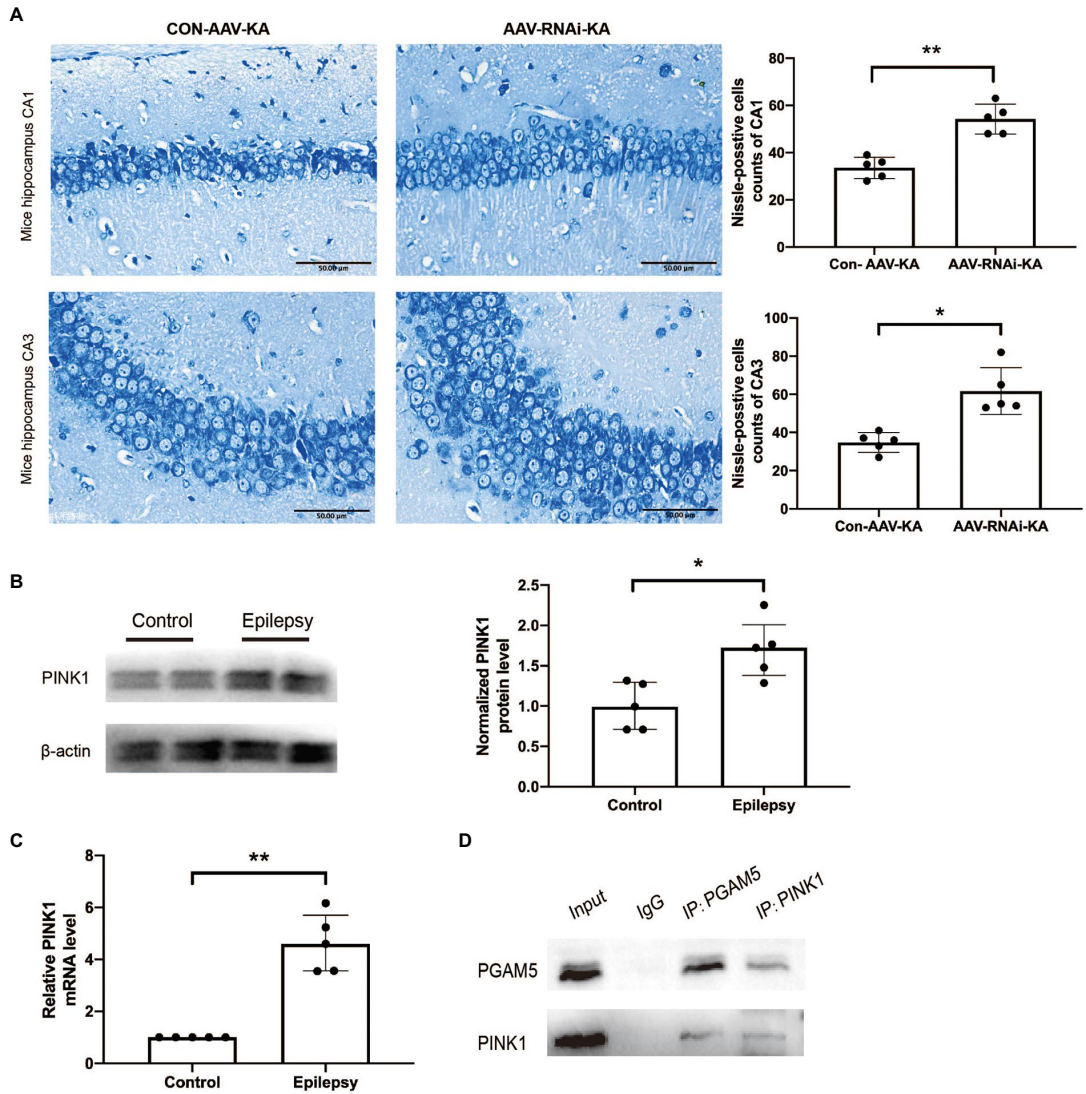
PGAM5 interacts with PINK1 in KA-induced mouse model and knockdown of PGAM5 inhibited mitophagy activity. **(A)** AAV-RNAi-KA group significantly increased the number of neuronal cell survival in the hippocampus after SE. Scale bar: 50 μm, *n* = 5. **(B)** Quantitative analysis of the PINK1/β-actin ratio, PINK1 was increased in the hippocampus of the KA-induced epilepsy model (*n* = 5). **(C)** Relative levels of PINK1 detected by RT-qPCR analysis (*n* = 5). Data are expressed as Median with interquartile range. **(D)** Co-immunoprecipitation between PGAM5 and PINK1 was demonstrated using anti-PGAM5 or anti-PINK1 antibodies.

In Con-AAV-KA, the neuronal cell structure in the mouse hippocampus was incomplete and ruptured. The contour and the boundary were blurred and unclear. The intercellular distance was increased, and the arrangement was disordered, and the chromatin clumping was observed at the edge. The cytoplasm was edematous, and fewer Nissl bodies could be found in it (Figure 4A). In summary, knockdown PGAM5 reduced the frequency and total time of seizures, and reduced the loss of neurons.

### PGAM5 interacted with PINK1 in KA-induced mouse model, and knockdown of PGAM5 attenuated PINK1 expression

**Figure 5:**
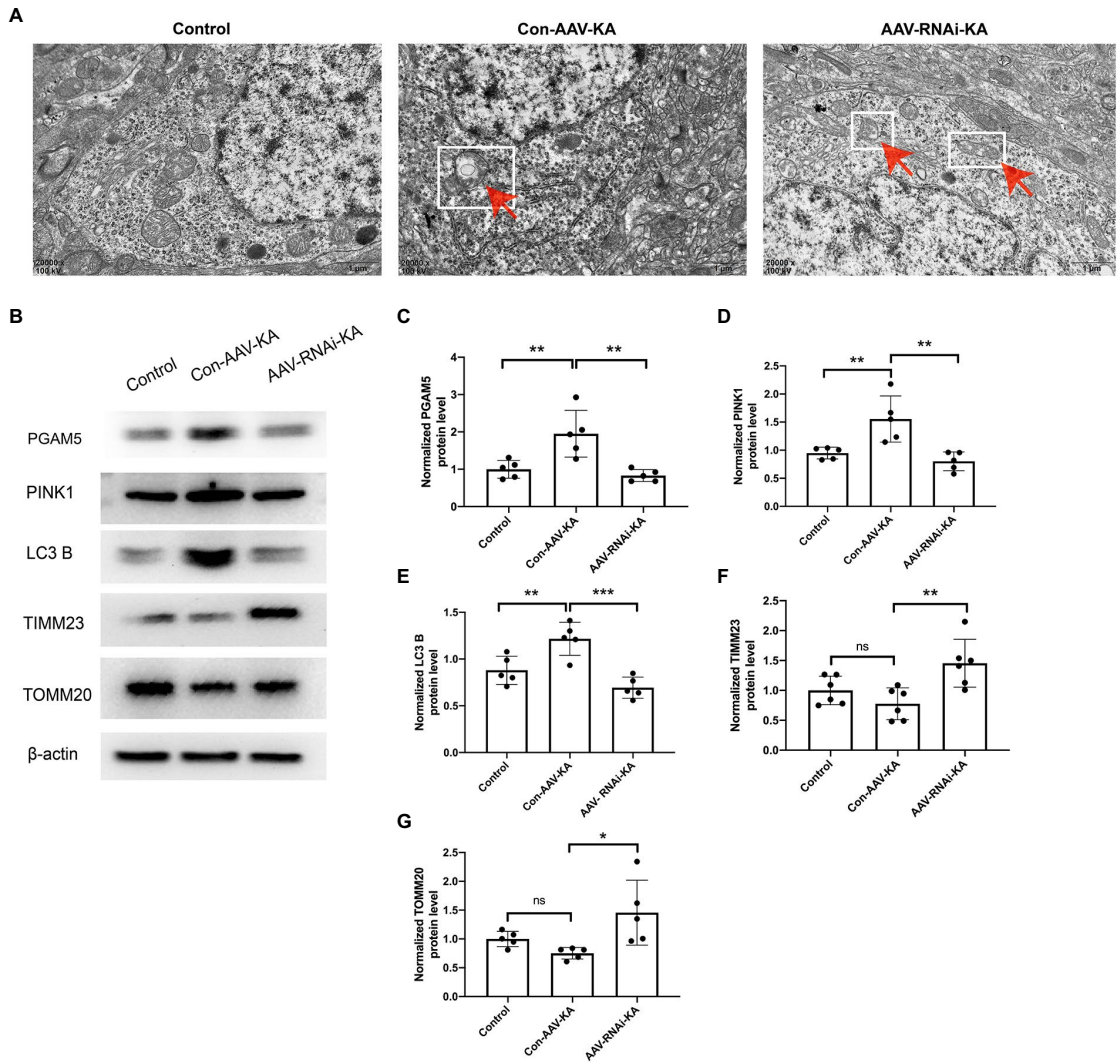
Knockdown of PGAM5 inhibited KA-induced mitophagy activity. **(A)** The representative images showed that the mitochondrial structure was normal in the Control group; mitochondria phagocytosed by double-membrane structures were labeled with red arrow and white square in the Con-AAV-KA group; no significant vacuolar degeneration and cell membrane rupture were observed in the AAV-RNAi-KA group by TEM. Scale bar: 1 μm. **(B–G)** Protein levels of PGAM5, PINK1, LC3 B, TIMM23, TOMM20 in the hippocampus of three groups were measured by western blot (*n* = 5). TEM, transmission electron microscope; PGAM5, phosphoglycerate mutase 5; PINK1, PTEN-induced putative kinase 1; LC3, light chain 3; TOMM20, translocase of the outer mitochondrial membrane 20; TIMM23, translocase of the inner mitochondrial membrane 23; IgG, immunoglobulin G. Data are expressed as Means ± SD *p < 0.05, **p < 0.01, ***p < 0.001, ****p < 0.0001.

Previous studies have found that PGAM5 may protect PINK1 from the degradation of mitochondria (Imai et al., 2010). However, it has not been specifically studied in epilepsy. Compared with normal control brain tissue, the PINK1 level in the brain tissue of KA-induced epileptic mice was significantly up-regulated (Figures 4B,C). The result of the co-immunoprecipitation assay showed that PGAM5 co-immunoprecipitated with PINK1 in the KA-model mouse (Figure 4D). Western blot results showed that PGAM5 expression was inhibited in AAV-RNAi-KA mice compared with the Con-AAV-KA group (Figures 5B,C). PINK1 protein levels in the Con-AAV-KA group were higher than that in the control group in Figures 5B,D. After injecting AAV, the PINK1 level was significantly lower than that in the Con-AAV-KA group. These data suggested that the knockdown of PGAM5 may have the ability to strongly reduce PINK1 expression in the KA-injected mice model.

### Knockdown of PGAM5 inhibited KA-induced mitophagy activity

To investigate the effect of PGAM5 on mitophagy, we selected electron microscopy to observe the morphology of neurocytes in the hippocampus. As shown in Figure 5A, clear and complete hippocampal neuron structure and the normal nuclear structure could be found in the control group. No double-membrane autophagosomes were detected. In the Con-AAV-KA group, apoptosis of most neuronal cells, shrunken nuclei, chromatin accumulation, rough endoplasmic reticulum, and mitochondrion swelling were clearly observed. Surprisingly, not only did the treatment with AAV make fewer neuronal cells undergo death but it also resulted in much mitochondrial morphology being restored. Besides, no significant vacuolar degeneration and cell membrane rupture were seen in the AAV-RNAi-KA group, which implies that neuronal morphology recovered after the knockdown of PGAM5. To further confirm these findings, protein expression of mitochondrial markers, including LC3 B (the autophagy-related marker), TIMM23 (an IMM protein), and TOMM20 (an OMM protein), was examined by Western blot. As shown in Figures 5B,E-G, compared with the Con group, LC3 B was increased, which was decreased after the AAV injection. The TIMM23 and TOMM20 protein expression were decreased in the Con-AAV-KA group, which was enhanced after AAV injection. Collectively, these data revealed that PGAM5 could reduce PINK1 expression to inhibit mitophagy activity during epilepsy after AAV intervention.

### Increased oxidative stress level in KA-induced SE and knockdown of PGAM5 alleviated MDA, Sod, and ROS levels

**Figure 6:**
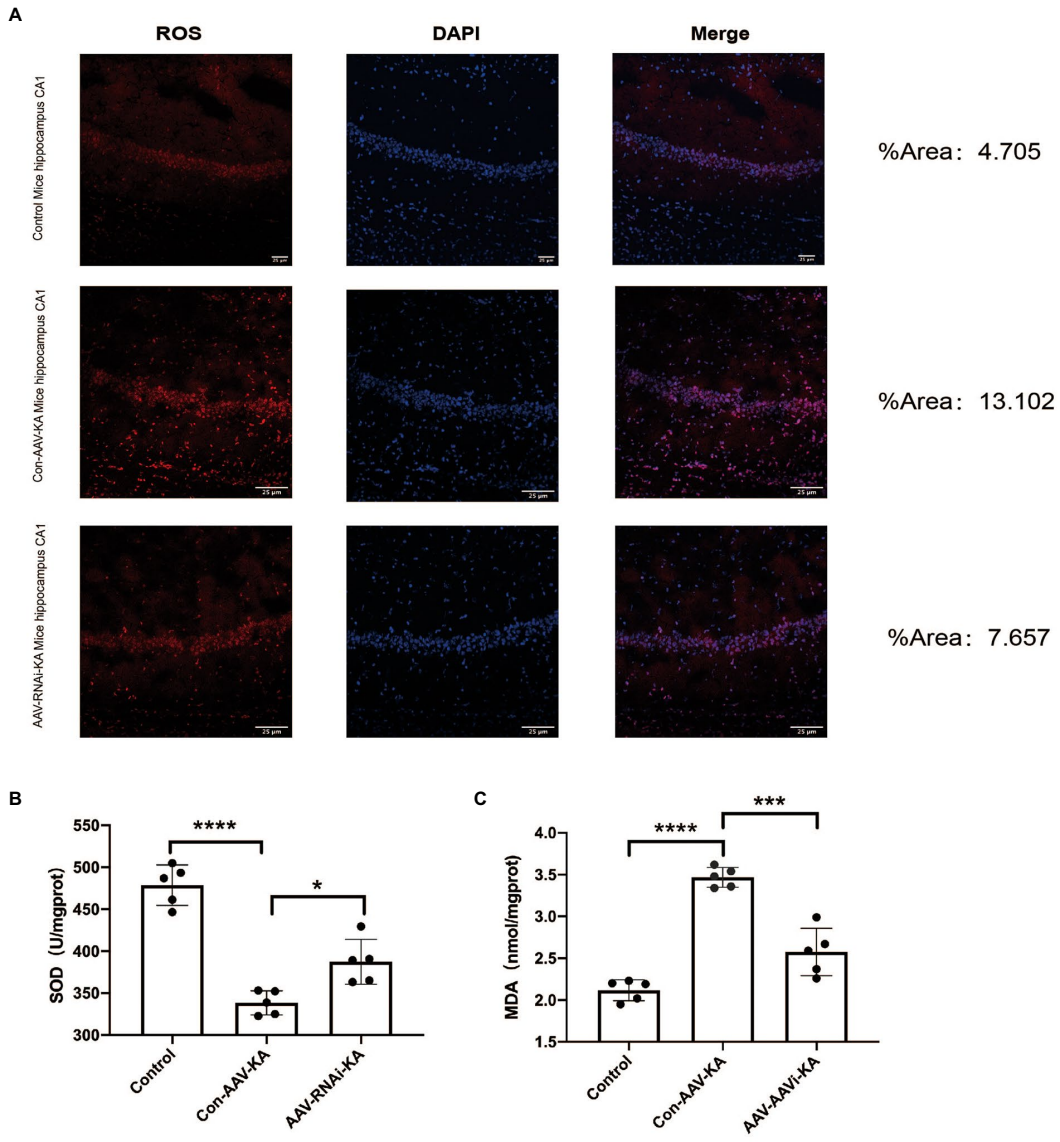
PGAM5 inhibition reduces ROS generation **(A)** Immunofluorescence staining of DHE expression in the hippocampus of mice in the Control, Con-AAV-KA and AAV-RNAi-KA groups (red). (*n* = 5) **(B,C)** Relative MDA and SOD contents in Control, Con-AAV-KA and AAV-RNAi-KA groups measured by the TBA and WST-8 methods. (*n* = 5); ROS, reactive oxygen species; DHE, dihydroethidium; MDA, malondialdehyde; SOD, superoxide dismutase; TBA, thiobarbituric acid; WST-8, water-soluble tetrazolium salt-8. Data are expressed as Means ± SD, *p < 0.05, ***p < 0.001, ****p < 0.0001.

To investigate the role of PGAM5 on oxidative stress in a KA-induced epilepsy model, we examined the ROS probe dihydroethidium (DHE) to detect ROS accumulation in mouse hippocampal neurons. The results showed that ROS generation and red fluorescence intensity were increased in the CA1, CA3, and DG regions of the hippocampus of mice in the Con-AAV-KA group. After the AAV injection, ROS generation was significantly attenuated (Figure 6A). Next, we examined oxidative stress by spectrophotometry. However, MDA content decreased and SOD activity increased after the AAV injection. Our results stated that oxidative stress could be attenuated after injection (Figures 6B,C).

## Discussion

The present study indicates that the expression of PGAM5 increases in neurons of epileptic mice, and increased mitophagy and ROS levels are also found in it. The knockdown of PGAM5 improves the ROS level, reduced neuronal damage and decreased the frequency of SLEs by inhibiting the PINK1-mediated mitophagy. More importantly, the interaction between PGAM5 and PINK1 is found in the epilepsy model. Therefore, we investigated the role of PGAM5 in the development and progression of epilepsy as an important molecule role in regulating mitochondrial homeostasis.

In our study, the increased PGAM5 level is found in the hippocampus of epileptic mice, which is mostly located in the CA1, CA3, and DG neurons of the hippocampus. Neuronal cell death is the main pathological outcome after seizures, thus the results suggest that PGAM5 may play an important role in epilepsy (de Lanerolle et al., 2003; Dong et al., 2013; Park et al., 2018). The pathophysiological changes in epilepsy include the weakening of protective factors to prevent seizures as well as the strengthening of damaging factors to induce seizures (Pitkänen and Lukasiuk, 2011). The inhibition of PGAM5 is considered as a protective effect based on the following evidence. The previous study has demonstrated that LFHP-1c, an inhibitor of PGAM5, plays a significant role in protecting cerebral vessels and maintaining blood–brain barrier function (Gao et al., 2021). In addition, some studies have also reported the inhibition of PGAM5 has a positive role in reducing neuroinflammation, rescuing mitochondrial function as well as improving oxidative stress (Johnson and Giulivi, 2005; Ooigawa et al., 2006; Chen et al., 2012). However, the role of PGAM5 in epilepsy remains unclear. Therefore, we explored the role of PGAM5 in seizures. Our results indicated that knockdown of PGAM5 reduced the frequency and total duration of seizures. This suggests that PGAM5 is a damaging factor based on the evidence presented above.

Next, we investigated whether PGAM5 is associated with mitophagy in a KA-induced epilepsy model. Previous studies have activated mitophagy participates in epilepsy development *via* mitochondrial dysfunction (Dillioglugil et al., 2010; Wu et al., 2018). Mitophagy in TLE is activated after mitochondrial damage, which leads to the clearance of mitochondria themselves (Wu et al., 2018). It has been reported that mitochondrial dysfunction in the CNS can not only lead to seizures, but also release harmful ROS and then result in neuronal degeneration or even death (Lin and Kang, 2010; Han et al., 2011; Sheng, 2014). Our results show that LC3 B level were significantly increased and TOMM20 and TIMM23 were significantly decreased in the epilepsy model, which indicates an enhanced level of mitophagy in our epileptic mice inducing by KA injection. However, reduced mitochondrial swelling and fragmentation are found after the inhibition of PGAM5, which suggests that the knockdown of PGAM5 is crucial for reducing mitophagy in epilepsy. In the previous study, the inhibition of mitophagy has a protective effect on acute neuronal injury (Cao et al., 2019; Gao et al., 2021). We revealed an increase in neuronal survival following PGAM5 knockdown. Therefore, according to these results, we speculated that PGAM5 might reduce neuron loss by inhibiting mitophagy.

Recent studies have shown that the hippocampus in the central nervous system (CNS) is vulnerable to abnormal mitophagy function by oxidative stress (Naziroğlu et al., 2008; Aguiar et al., 2013; Wu et al., 2018). Our results suggest that the aggravated oxidative damage, increased ROS as well as MDA contents and attenuated SOD activity in the epilepsy model. However, these results were reversed by PGAM5 inhibition. Moreover, decreased SOD levels are discovered in human epileptic cerebrospinal fluid, especially in patients with refractory epilepsy (Chen et al., 2012). Also, increasing fragile mitochondria, mitochondrial dysfunction and oxidative stress are found in a variety of animal models of acquired epilepsy (e.g., KA-induced epilepsy model, PTZ-induced epilepsy model, pilocarpine-induced epilepsy model) (Liang et al., 2000; Jarrett et al., 2008; Ryan et al., 2012). This demonstrates that the generation of ROS associated with mitochondrial defects can also affect epileptogenesis (Shekh-Ahmad et al., 2019). The oxidative stress during seizures may cause mitochondrial damage. Notably, ASDs with antioxidant properties have been proven to have neuroprotective effects (Bashkatova et al., 2003; Willmore, 2005). Our study suggests that the reduction in neuronal loss after the knockdown of PGAM5 is attributed to the attenuation of oxidative stress.

The PINK-mediated mitophagy pathway plays an important role in Parkinson’s disease, aging rats, SH-SY5Y neuroblasts, cerebral ischemia–reperfusion injury (CIRI), and Alzheimer’s disease (Pickrell and Youle, 2015; Martín-Maestro et al., 2016; Park et al., 2018; Yi et al., 2019; Ma et al., 2021; Wen et al., 2021; Mao et al., 2022). PINK1 expression is increased in our epilepsy model and decreased PINK1 levels are detected in the epilepsy model after inhibition of PGAM5. The co-immunoprecipitation shows the interaction between PGAM5 and PINK1, which suggests that PGAM5 may be involved in the PINK1-mediated mitophagy pathway. In previous studies, PGAM5 promote PINK1-mediated mitophagy and stabilized PINK1 on OMM (Sugo et al., 2018). Accordingly, PINK1 degradation induced by PGAM5 depletion causes a failure to initiate mitophagy. This suggests that PINK1 may be critical for increased mitophagy caused by PGAM5 in epilepsy. All these studies reveal that PGAM5 may regulate mitophagy in epilepsy *via* PINK1.

**Figure 7:**
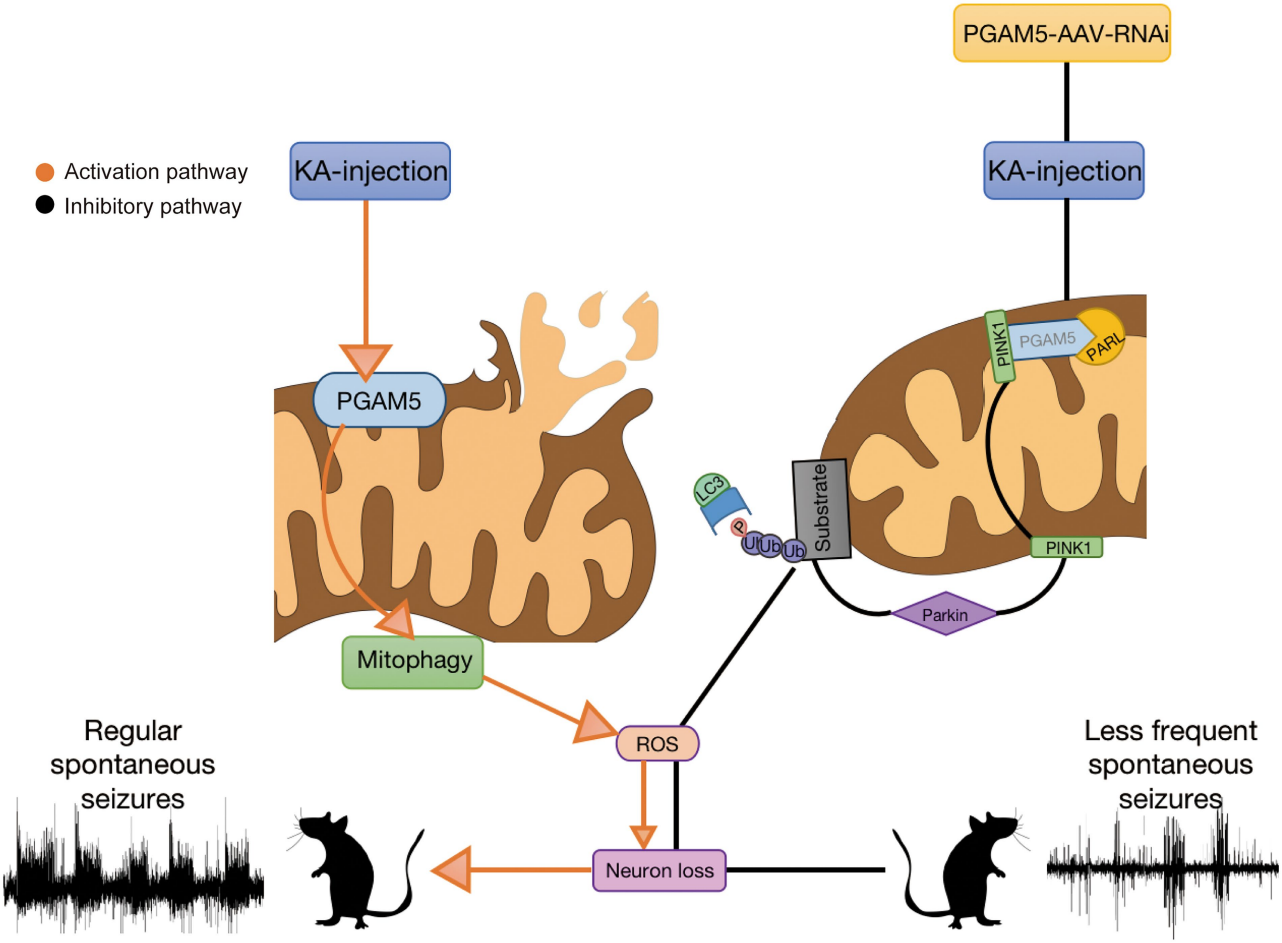
Seizures induce excessive activation of mitophagy and lead to oxidative stress. PGAM5 knockdown resists reactive oxygen species accumulation and protects neurons from mitophagy.

Herein, we address the possible mechanism of PGAM5 in epilepsy based on our study. During the progression of epilepsy, increased PGAM5 maybe contributes to mitochondrial damage. Meanwhile, mitophagy may further lead to excessive activation of oxidative stress. ROS production induces the enhancement of mitophagy and aggravates neuronal injury, which results in severe consequences including massive loss of epileptic neurons and increased SLEs (Tizon et al., 2010). After injection of AAV, rhomboid-like protein (PARL) directly combines with PINK1 based on inhibition of PGAM5, and degenerates PINK1 accumulation in IMM (Dillioglugil et al., 2010). The inhibited PINK1 transport leads to the accumulation of PINK1 on the OMM. Accumulated PINK1 ubiquitinates OMM proteins by recruiting Parkin to damaged mitochondria, and finally causing a failure in the initiation of PINK1-mediated mitophagy (Imai et al., 2010; Lu et al., 2014; Park et al., 2018). The process ultimately leads to reducing PINK1-mitophagy. Following decreased PGAM5-PINK1-mediated mitophagy, subsequent attenuation of ROS and increased neuronal survival are demonstrated. Finally, sustained epileptic activity is significantly reduced. Our study suggests that the knockdown of PGAM5 has a significant attenuating effect on epilepsy *via* mitophagy (Figure 7). Therefore, PGAM5 is a potential target for epilepsy therapy.

The knockdown efficiency of AVV-RNAi has been confirmed in our experimental results, but the off-target effect of AVV failed to be completely excluded after detection. Therefore, more rigorous experiments may be needed to confirm it. Besides, PGAM5 is significantly up-regulated in the late stage of epilepsy. Considering that most of the neurons had died in this period, it may be necessary to find surviving neurons in epilepsy models to rule out the effect of dead neurons on PGAM5 expression. Lastly, although our results proved that PGAM5 can induce epileptogenesis by promoting PIN1-mediated mitophagy, the indirect effect of PGAM5 on PINK1-mediated mitophagy cannot be ruled out. Further work will be needed to verify this conjecture.

## Conclusion

In conclusion, our findings report the unnoticed but important role of PGAM5 in epilepsy. We report that the knockdown of PGAM5 inhibits mitophagy, attenuates seizures, and improves neuronal survival in a KA-induced epilepsy model. These findings contribute to our understanding of the role of PGAM5 in epilepsy and provide a novel target for the treatment of epilepsy.

## Data availability statement

The original contributions presented in the study are included in the article/Supplementary material, further inquiries can be directed to the corresponding author.

## Ethics statement

The animal study was reviewed and approved by the Commission of Chongqing Medical University.

## Author contributions

FZ was a major contributor in performing experiments and writing the manuscript. YG carried out the operation of tissue and related experiments. JS, WZ, and SY performed the tissue collection and analysis of the data. JW provided ideas for the experiments. YL and WY designed the study and revised the manuscript. All authors read and approved the final manuscript.

## Funding

This study was supported by grants from the National Natural Science Foundation of China (81871019 and 81671286). Chongqing Postgraduate Scientific Research Innovation Project (CYS20188).

## Conflict of interest

The authors declare that the research was conducted in the absence of any commercial or financial relationships that could be construed as a potential conflict of interest.

## Publisher’s note

All claims expressed in this article are solely those of the authors and do not necessarily represent those of their affiliated organizations, or those of the publisher, the editors and the reviewers. Any product that may be evaluated in this article, or claim that may be made by its manufacturer, is not guaranteed or endorsed by the publisher.

## Footnotes

^1^ https://blast.ncbi.nlm.nih.gov/Blast.cgi

## Abbreviations

PGAM5: Phosphoglycerate mutase 5
PINK1: PTEN-induced putative kinase 1
ASDs: antiseizure drugs
KA: kainic acid
TLE: temporal lobe epilepsy
AAV: Adeno-associated virus
DG: dentate gyrus
SPF: specific pathogen-free
TOMM20: translocase of the outer mitochondrial membrane 20
TIMM23: translocase of the inner mitochondrial membrane 23
LC3 B: light chain 3 B
MAP2: microtubule-associated protein 2
GFAP: glial fibrillary acidic protein
IBA1: ionized calcium-binding adapter molecule 1
TBST: tris-buffered saline with Tween-20
PBST: pbs saline containing Tween-20
MDA: malondialdehyde
SOD: superoxide dismutase
ROS: reactive oxygen species
TEM: transmission electron microscope
RT-qPCR: Reverse transcription-quantitative polymerase chain reaction
LFPs: local field potentials
SE: status epilepticus
SLEs: spontaneous seizure-like events
OMM: outer mitochondrial membrane
IMM: inner mitochondrial membrane
DHE: dihydroethidium
OS: oxidative stress
PARL: presenilin-associated rhomboid-like protein
