# Supplementary material for: The inhibition of PGAM5 suppresses seizures in a kainate-induced epilepsy model via mitophagy reduction
Source: Front Mol Neurosci. 2022 Dec 22;15:1047801. doi: 10.3389/fnmol.2022.1047801 (PMC9813404; doi:10.3389/fnmol.2022.1047801)
Supplement: Supplementary file 1 [file Data_Sheet_1.docx]

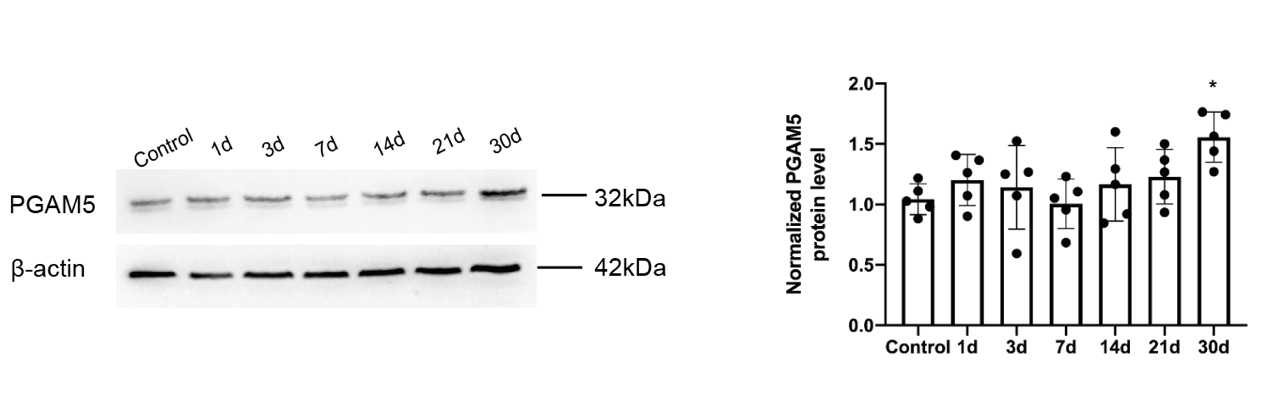


Supplement Fig.1 Expression of PGAM5. Compared with control group, PGAM5 was most highly expressed at 30 days in hippocampus in the KA-induced TLE model.


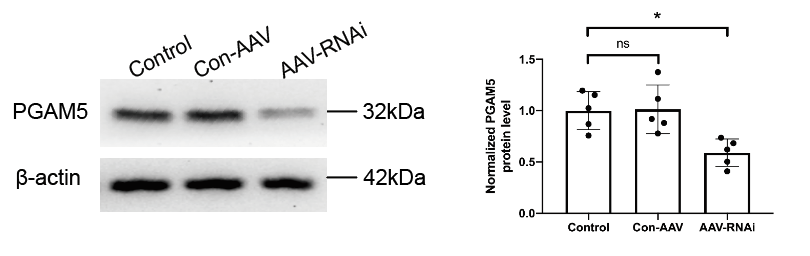


Supplement Fig.2 Knockdown PGAM5 efficiency. Compared to the control injection, the knockdown efficiency of PGAM5 was 58%.
